# How Predictable Are the Behavioral Responses of Insects to Herbivore Induced Changes in Plants? Responses of Two Congeneric Thrips to Induced Cotton Plants

**DOI:** 10.1371/journal.pone.0063611

**Published:** 2013-05-14

**Authors:** Rehan Silva, Michael J. Furlong, Lewis J. Wilson, Gimme H. Walter

**Affiliations:** 1 School of Biological Sciences, The University of Queensland, St Lucia, Queensland, Australia; 2 Cotton Research Unit, CSIRO Plant Industry, Narrabri, New South Wales, Australia; French National Institute for Agricultural Research (INRA), France

## Abstract

Changes in plants following insect attack are referred to as induced responses. These responses are widely viewed as a form of defence against further insect attack. In the current study we explore whether it is possible to make generalizations about induced plant responses given the unpredictability and variability observed in insect-plant interactions. Experiments were conducted to test for consistency in the responses of two congeneric thrips, *Frankliniella schultzei* Trybom and *Frankliniella occidentalis* Pergrande (Thysanoptera: Thripidae) to cotton seedlings (*Gossypium hirsutum* Linneaus (Malvales: Malvaceae)) damaged by various insect herbivores. In dual-choice experiments that compared intact and damaged cotton seedlings, *F. schultzei* was attracted to seedlings damaged by *Helicoverpa armigera* (Hübner) (Lepidoptera: Noctuidae), *Tetranychus urticae* (Koch) (Trombidiforms: Tetranychidae), *Tenebrio molitor* Linnaeus (Coleoptera: Tenebrionidae), *F. schultzei* and *F. occidentalis* but not to mechanically damaged seedlings. In similar tests, *F. occidentalis* was attracted to undamaged cotton seedlings when simultaneously exposed to seedlings damaged by *H. armigera*, *T. molitor* or *F. occidentalis*. However, when exposed to *F. schultzei* or *T. urticae* damaged plants, *F. occidentalis* was more attracted towards damaged plants. A quantitative relationship was also apparent, *F. schultzei* showed increased attraction to damaged seedlings as the density of *T. urticae* or *F. schultzei* increased. In contrast, although *F. occidentalis* demonstrated increased attraction to plants damaged by higher densities of *T. urticae*, there was a negative relationship between attraction and the density of damaging conspecifics. Both species showed greater attraction to *T. urticae* damaged seedlings than to seedlings damaged by conspecifics. Results demonstrate that the responses of both species of thrips were context dependent, making generalizations difficult to formulate.

## Introduction

Insect herbivores are diverse and feed on plants in various ways. Plants respond biochemically to herbivore, and pathogen, attack [1], [2] and these induced responses are widely considered to constitute a form of defence [3], [4]. The volatiles released by plants in response to insect herbivory alter the course of the insect-plant interaction [5], [6] and reduced rates of attraction of certain herbivores to damaged plants are often cited as evidence of a defensive role for these chemicals [1], [3], [4]. Herbivore-induced plant interactions become more intricate when herbivore natural enemies are also considered. The biochemical changes induced in plants have been interpreted as indirect defences as they can attract predators and parasitoids of the damaging herbivores and they are presumed to ultimately increase the fitness of the induced plant [7]. The importance of induced plant responses is no longer controversial [8] but the function of these responses remains a question for debate. Results from specific studies have frequently been used to support functional interpretation of induced responses including their ecological impact, their effect on the fitness of the impacted herbivores and even possible coevolutionary relationships between plants and the natural enemies of their herbivores [9]. It thus begs the question: under what circumstances can we make generalizations about induced plant responses to insect herbivory? Simply put, can we extrapolate from specific studies to draw broad scale conclusions about induced plant responses, or are conclusions from specific studies only valid to the particular systems studied?

It is important to acknowledge that the majority of examples of induced ‘resistance’ have been measured from the perspective of the herbivore and there is little evidence of increased plant fitness, and therefore that the response should be considered a defence [8], [7], [10]. For instance, herbivores that feed on induced plants may take longer to complete development and consume more tissue than insects feeding on non-induced plants; consequently they may be more damaging to plants than herbivores that grow more quickly but consume less plant material [8]. In addition, with regard to the interaction between natural enemies and plants, there is no proof that the recorded attraction of natural enemies actually leads to the suppression of attacking herbivore populations [10]. Further, most parasitoids do not immediately kill their hosts, which means that plant damage continues for some time after herbivores have been attacked by the attracted parasitoid natural enemies [7]. Whether herbivores induce specific volatiles that attract specific natural enemies is also debatable; for example, *Microplitis croceipes* (Cresson) (Hymenoptera: Braconidae), which attacks the corn earworm (*Helicoverpa zea* (Boddie) (Lepidoptera: Noctuidae)), was equally attracted to plants treated with regurgitant of the beet armyworm, *Spodoptera exigua* (Hübner) (Lepidoptera: Noctuidae) (which it does not attack), as it was to plants treated with *H. zea* regurgitant [10]. In short there are many factors that determine the outcomes of insect-plant interactions and the observed measurable phenomena in one system may not be evident in others.

In general, induced responses tend to negatively affect the oviposition and attractancy responses of adult lepidopterans [11]–[16], yet there is considerable variability in the responses of insects to damaged plants more generally. In some cases induced responses have lead to an increase in insect oviposition and attractancy responses [12], [17]–[20], and the plants involved become more susceptible to additional attack by herbivores [8]. For example, the diamondback moth (*Plutella xylostella* L. (Lepidoptera: Plutellidae)), showed varying oviposition responses to three species of damaged *Brassica* plants; on both cabbage and canola oviposition was greater on damaged plants than on undamaged plants, but on Chinese cabbage oviposition was greater on undamaged plants [12]. Thus host plant responses to herbivory can be unpredictable and the measurement of one response by a given insect to herbivore induced changes in the plant is unlikely to define the insect-plant relationship. In the view of such variability it is worth considering just how much can be generalized about induced plant responses and their evolutionary ecology.

In this study, the interactions between two congeneric thrips species, *Frankliniella schultzei* Trybom and *F. occidentalis* Pergande (Thysanoptera: Thripidae), and cotton (*Gossypium hirsutum*), a host plant common to both, were investigated for consistency. By feeding on the cotyledons and the developing terminals of young cotton seedlings [21], these thrips cause damage that can sometimes result in yield loss or delayed maturity, though often plants are able to compensate [22], [23]. Hence, given evidence of induced responses in cotton to other herbivore species [24]–[26], we might predict that both species of thrips would respond similarly.

Both *F. schultzei* and *F. occidentalis* have an additional aspect to their interaction with plants. Neither fits neatly into the ‘herbivore’ category as both species also consume eggs of spider mites, and their feeding can delay the development of mite outbreaks [27]–[29]. Indeed, mite eggs provide a valuable dietary component in relation to cotton as both thrips species have improved reproductive output if they have access to mite eggs on cotton leaf tissue [30], [31]. Predictions about behaviour need to take account of these intricacies. We might expect, for example, that both species of thrips should be attracted more strongly to cotton seedlings that have been damaged by mites, assuming that the value of mite eggs in the diet outweigh any negative effects of induced responses in the plant tissue. This further assumes that thrips can detect differences between plants damaged by mites or by other herbivores. Mite damaged cotton is attractive to *F. occidentalis* [29], but no such information is yet available for *F. schultzei*.

In this study we tested for consistency in the responses of these two congeneric *Frankliniella* thrips to mechanical damage, damage induced by various herbivores and damage caused by potential prey. The study aimed to determine specifically (1) whether damage inflicted by arthropods with different modes of feeding affected the responses of thrips in similar ways, (2) whether the degree of damage affected subsequent attractiveness to thrips, (3) the effect of mite damage as opposed to thrips damage on the attractiveness to thrips, (4) how *F. schultzei* responds to *F. occidentalis* damaged cotton seedlings and (5) how *F.* *occidentalis* responds to *F. schultzei* damaged cotton seedlings.

## Materials and Methods

### Ovipositional and Attractancy Responses of Arthropod Herbivores to Induced Host Plants – Literature Search

The Science Citation Index Expanded in the Thomson Reuters Web of Science® database was searched using the advanced search function. The field tags and search terms TS = (herbivore* SAME oviposition) and TS = (herbivore* SAME insect attraction*) were used to search for journal articles published in English from 1990–2012. Output was scrutinized for studies which investigated the effects of host plant induction (by herbivore feeding, application of elicitors or mechanical damage).

### Plants

Cotton plants (*G. hirsutum* var. 71RRF) were raised from seed in seed trays containing organic potting mix supplemented with slow-release fertilizer [Osmocote (N:P:K, 16∶35:10); Scotts Australia, Baulkham Hills, New South Wales]. Five days after germination, seedlings were transplanted individually into pots (11 cm diameter) with the same mixture of organic potting mix and fertilizer; they were watered daily and grown under natural light and temperature conditions in a ventilated greenhouse. Plants were used in experiments when they were at the two-leaf stage.

### Insects

*Frankliniella schultzei* and *F. occidentalis* used in all experiments were from a laboratory culture that originated from adults collected from the field. *Frankliniella schultzei* were collected from *Malvaviscus arboreus* Cav. (Malvales: Malvacea) flowers at the St Lucia campus of The University of Queensland, in Brisbane, Queensland, Australia. *Frankliniella occidentalis* were either collected from clover *Trifolium repens* L. (Fabales: Fabaceae), at the Gatton Research Station, Gatton, Queensland, or from cotton (*G. hirsutum*) flowers at the Australian Cotton Research Institute at Myall Vale near Narrabri, New South Wales. Insects were reared in glass jars (17 cm in height, 8 cm diameter) held in an incubator (25±2°C, L12:D12). To begin each culture, fresh green beans (*Phaseolus vulgaris*) were soaked in water for 16 h and then washed in warm soapy water to remove any insecticide residue. They were rinsed in clean water and dried before being used as a rearing substrate for the thrips. Cleaned beans were placed in glass jars lined with paper towel and female thrips were released to feed and oviposit. To prevent thrips from escaping, glass jars were sealed using two layers of nylon mesh (22×22×22 cm, ≈1 mm^2^) with a black cardboard layer held between them. The green beans were left in the glass jars for three days after which they were removed and replaced with new ones; old beans were placed in new glass jars lined with paper towel for larval rearing. For both adult and larval thrips, pollen collected from *Hibiscus* spp (Malvales: Malvaceae) flowers was provided as an additional food supplement. All experiments were conducted on lab reared thrips 3–5 generations after field collection.

### Standard Olfactometer Tests

The same method was used in all olfactometer experiments. A clean glass Y-tube olfactometer (stem 9 cm; arms 9 cm each at a 45° angle; internal diameter 0.9 cm) was used to compare the responses of thrips in various dual-choice experiments. Air was drawn over an activated charcoal filter and then through the olfactometer at a rate of 1 mL min^−1^. Experiments were conducted in a temperature controlled room at 25°C (±2°C), and a light source was placed directly above the Y junction of the olfactometer to avoid bias. Single female thrips were released at the stem entrance of the Y-tube and observed for 10 min. When a single thrips moved more than half way (4.5 cm) into one of the arms and remained there for more than 20 s, it was recorded as being attracted to the odour source associated with that arm. After testing five individuals, the chambers with plants were rotated and the Y- tubes cleaned with 100% ethanol and dried. After 10 thrips individuals had been screened, the test plants were changed. A minimum of 60 individual thrips was used in each test.

### Standard Method for Setting up Arthropod Enclosures

Cotton seedlings for use in olfactometer experiments were either damaged mechanically or by *H. armigera* larvae, adult thrips, two spotted spider mites (*T. urticae*) or mealworms (*T. molitor*) (see below for details). Cages were constructed to enclose the arthropods used to damage the potted cotton seedlings. All arthropods were enclosed on potted cotton seedlings within inverted plastic cups (9.7 cm in height, 8 cm diameter) containing two windows (3.5×4.5 cm) covered with nylon mesh (≈1 mm^2^). Once the relevant arthropods had been placed inside the cup, the rim was pushed onto the soil surface to enclose them on the seedling and they were allowed to feed for 24 h.

### Feeding Styles, Cotton Responses and Thrips Behaviour

A series of dual-choice olfactometer experiments was conducted to examine thrips behaviour in response to cotton seedling damage. In each experiment the response of thrips to damaged cotton seedlings and undamaged cotton seedlings was compared. The following forms of damage were inflicted on cotton seedlings:

1. **Mechanical damage.** Both leaves of the cotton seedlings were damaged mechanically in a manner designed to simulate the feeding damage of various caterpillars that attack cotton plants. Circular holes (0.5 cm diameter) were cut through leaf tissue using a cork borer. A total of six holes (three on each leaf) were cut. Plants were damaged 24 h before the start of the standard olfactometer experiments.
2. ***H. armigera*** **damage**. Cotton leaves were damaged by second instar *H. armigera* larvae. Ten larvae were introduced to the adaxial leaf surface and were enclosed as previously described. Larvae were allowed to feed on the seedlings for 24 h. Standard olfactometer experiments were performed using plants with *H. armigera* larvae *in situ* and with plants from which *H. armigera* larvae were removed after 24 hrs feeding.
3. **Thrips damage.** Cotton foliage was independently damaged by *F. schultzei* and *F. occidentalis*. Fifty thrips (densities of this magnitude have been recorded in the field [32]) were introduced onto each plant and were enclosed as described above. Thrips were allowed to feed on seedlings for 24 h. Standard olfactometer experiments were performed using plants with thrips *in situ* or with plants from which thrips were removed after 24 hrs feeding.
4. **Mite damage.** Fifty mites (densities of this magnitude have been recorded in the field [32]) were introduced onto the leaves and were enclosed as described above. Mites were allowed to feed on cotton seedlings for 24 h. Standard olfactometer experiments were performed using plants with mites *in situ* and with plants from which mites were removed after 24 hrs feeding.
5. **Root damage.** Roots of potted cotton seedlings were damaged by using mealworms (*T. molitor*). These cause damage similar to that of false wireworm species (also Tenebrionidae, and which are pests of cotton [32]). They damage roots by feeding or by dislodging them as they burrow. Ten mealworms were placed on the soil surface and allowed to burrow into the soil. The response of thrips to root-damaged and undamaged above ground foliage was measured in standard olfactometer experiments 48 h after release of the mealworms whereupon damage began to be inflicted. After the olfactometer experiments the cotton seedlings were removed from the pots and the mealworms extracted. Each cotton seedling was placed in a plastic box (45×25×10 cm) and exposed to slow running water. The root mass was removed from the stem of each plant and all broken and dislodged pieces of root in the soil were collected. All root material was then dried in an oven at 60°C for 24 h. The dried root mass was then weighed and the number of dislodged pieces of root recorded before this material was pooled and weighed independently of the root mass. Root herbviory reduced root biomass by an average of 43%.

### Degree of Foliage Damage and Thrips Attraction

To determine if a quantitative relationship was detectable between the degree of damage and subsequent thrips attraction, experiments were conducted in which thrips preference was compared between plants that had been subjected to a high degree of herbivory and plants that had been subjected to a much lower degree of herbivory. The arthropods used to damage cotton seedlings were mites and thrips. These arthropods were independently enclosed on cotton seedlings as previously described and allowed to feed for 24 h when they were removed and standard olfactometer experiments performed. For the thrips-damaged plant tests, plants damaged by 50 adult thrips and plants damaged by 10 adult thrips were compared. Similarly, for mite-damaged tests, plants damaged by 50 mites and plants damaged by 10 mites were also compared.

### Mite Damage vs Thrips Damage on Thrips Attractancy

The relative attraction of thrips to mite-damaged plants and thrips-damaged plants was investigated by exposing cotton seedlings to either 50 mites or to 50 thrips in insect enclosures for 24 h as previously described. Standard olfactometer experiments were performed using plants with herbivores *in situ* and with plants from which herbivores were removed after 24 hrs feeding.

### Are Thrips Attracted to Mites alone (No Plants) or a Combination of Mites and Damaged Plants?

Experiments were conducted to determine whether thrips were attracted to mite odors or whether the mites had to be present with damaged plants for thrips to respond. To establish if thrips were attracted to mite odours alone 50 mites were placed in one olfactometer chamber and the other was left empty. Separate standard olfactometer tests were then performed to investigate the attraction of *F. schultzei* and *F. occidentalis* to mites. In a second experiment cotton seedlings were exposed to 50 mites for 24 h in arthropod enclosures as previously described, mites were then removed from some plants but left *in situ* on others and standard olfactometer tests performed to test the attraction of *F. schultzei* and *F. occidentalis* to mite-infested and mite-damaged plants from which mites had been removed.

### Response of each *Frankliniella* Species to Cotton Seedlings Damaged by the other *Frankliniella* Species

Cotton seedlings were exposed to either 50 *F. schultzei* adults or 50 *F. occidentalis* adults for 24 h in arthropod enclosures as previously described. The thrips were then removed and standard olfactometer tests performed to test the attraction of *F. schultzei* to plants damaged by *F. occidentalis* relative to undamaged plants and the attraction of *F. occidentalis* to plants damaged by *F. schultzei* relative to undamaged plants.

### Statistical Analysis

The number of thrips attracted to the different treatments in paired olfactometer tests was compared by a statistical χ^2^ test using StatView [33]. Responses were converted to percentages for presentation.

## Results

### Ovipositional and Attractancy Responses of Arthropod Herbivores to Induced Host Plants – Literature Search

We found 37 published studies that examined how plant damage affected the attraction and oviposition responses of insect herbivores (Table 1). Of the studies that investigated the effect of plant induction on oviposition responses (n = 30), 33% recorded an increase in oviposition on induced plants, 63% recorded a decrease and 33% recorded that plant induction had no effect (Table 1). Similarly, for attractancy responses (n = 10), 60% recorded an increase on induced plants, 70% recorded a decrease and 40% recorded no effect (Table 1).

**Table 1:** Ovipositional and attractancy responses of various arthropod herbivores to host plants that have been “induced” in various ways.

Behavioral response	Mode of induction	Host plant	Herbivore		reference
			order	species	
**Oviposition**					
**Increased**	HC-F	*Brassica oleracea*	Lepidoptera	*Plutella xylostella*	[12], [17], [18], [19], [43]
		*Lycopersicon esculentum*		*Spodoptera exigua*	[20]
		*Gossypium hirsutum*		*Spodoptera littoralis*	[24]
		*Raphanus raphanistrum*		*Pieris rapae*	[4]
	MD-F	*Brassica oleracea*		*Plutella xylostella*	[12]
	JA				[19]
	MD-F, MD-R	*Brassica napus*			[12]
	HS-F	*Solanum tuberosum*	Diptera	*Episyrphus balteatus*	[44]
		*Phaseolus vulgaris*	Thysanoptera	*Frankliniella occidentalis*	[34]
**Decreased**	HC-F	*Solanum carolinense*	Coleoptera	*Leptinotarsa juncta*	[45]
		*Solanum dulcamara*		*Plagiometriona clavata*	[46], [47]
		*Mimosa pigra*		*Coelocephalapion aculeatum*	[48]
		*Sasa nipponica*	Diptera	*Procystiphora uedai*	[49]
		*Nicotiana attenuata*	Lepidoptera	*Manduca quinquemaculata*	[11]
		*Nicotiana tabacum*		*Heliothis virescens*	[50]
		*Lycopersicon esculentum*		*Helicoverpa armigera*	[13]
		*Zea mays*		*Ostrinia furnacalis*	[51]
	MeJA				[11]
	JA	*Capsicum annuum*	Diptera	*Liriomyza trifolii*	[52]
		*Brassica oleracea*	Lepidoptera	*Pieris rapae*	[14]
				*Pieris brassicae*	[14]
		*Arabidopsis*	Thysanoptera	*Frankliniella occidentalis*	[53]
		*Brassica rapa*			[53]
	HC-R	*Brassica nigra*	Lepidoptera	*Pieris brassicae*	[54]
		*Gossypium hirsutum*		*Spodoptera littoralis*	[25]
	HC-F, MD-F	*Brassica rapa*	Coleoptera	*Phaedon cochleariae*	[55]
		*Lycopersicon esculentum*	Lepidoptera	*Spodoptera exigua*	[20]
		*Brassica oleracea*		*Mamestra brassicae*	[18]
		*Nicotiana tabacum*	Thysanoptera	*Frankliniella occidentalis*	[56]
	MD-F	*Brassica rapa*	Lepidoptera	*Plutella xylostella*	[12]
	HS-F	*Solanum lycopersicum*	Diptera	*Liromyza trifolii*	[57]
	HC-F, JA	*Brassica campestris*		*Plutella xylostella*	[19]
**No effect**	HC-F	*Lythrum salicaria*	Coleoptera	*Hylobius transverovittatus*	[58]
		*Brassica oleracea*	Lepidoptera	*Pieris rapae*	[17], [18]
		*Solanum lycopersicum*	Hemiptera	*Bemisia argentifolli*	[57]
		*Triticum aestivum*	Hymenoptera	*Cephus cinctus*	[59]
	HC-R			*Galerucella calmariensis*	[58]
		*Brassica nigra*	Lepidoptera	*Pieris rapae*	[54], [60]
			Neuroptera	*Chrysoperala carnea*	[60]
	MD-F	*Brassica rapa*	Coleoptera	*Phaedon cochleariae*	[55]
		*Raphanus raphanistrum*	Lepidoptera	*Pieris rapae*	[4]
	MD-R	*Brassica rapa*		*Plutella xylostella*	[12]
**Attraction**					
**Increased**	HC-F	*Capsicum annuum*	Coleoptera	*Anthonomus eugenii*	[61]
		*Malus domestica*	Lepidoptera	*Cydia pomonella*	[15]
	JA	*Brassica oleracea*		*Plutella xylostella*	[19]
	HS-F	*Gossypium hirsutum*	Thysanoptera	*Frankliniella occidentalis*	[35]
	HC-F, MD-F	*Brassica oleracea*	Lepidoptera	*Mamestra brassicae*	[16]
		*Gossypium hirsutum*		*Trichoplusia ni*	[26]
**Decreased**	MD-F, HC-F	*Zea mays*	Hemiptera	*Rhopalosiphum maidis* (winged)	[62]
	HC-F	*Nicotiana tabacum*	Lepidoptera	*Heliothis virescens*	[50]
		*Brassica oleracea*		*Trichoplusia ni*	[26]
		*Zea mays*	Homoptera	*Cicadulina storeyi*	[63]
	MD-F	*Nicotiana tabacum*	Thysanoptera	*Frankliniella occidentalis*	[56]
	JA	*Brassica campestris*	Lepidoptera	*Plutella xylostella*	[19]
	HS-F	*Gossypium hirsutum*	Thysanoptera	*Frankliniella occidentalis*	[35]
**No effect**	MD-F, HC-F	*Zea mays*	Hemiptera	*Rhopalosiphum maidis* (wingless)	[62]
	MD-F	*Malus domestica*	Lepidoptera	*Cydia pomonella*	[15]
		*Brassica oleracea*		*Trichoplusia ni*	[26]
		*Solanum lycopersicum*		*Mamestra brassicae*	[16]
	HC-F	*Brassica oleracea*			[16]

Modes of induction were; herbivore chewing on foliage (HC-F), herbivore chewing on roots (HC-R), herbivore sucking on foliage (HS-F), mechanical damage to foliage (MD-F), mechanical damage to roots (MD-R) and the application of the biochemical elicitor jasmonic acid (JA) and methyl jasmonate (MeJA).

### Feeding Styles, Cotton Responses and Thrips Behaviour

**Figure 1:**
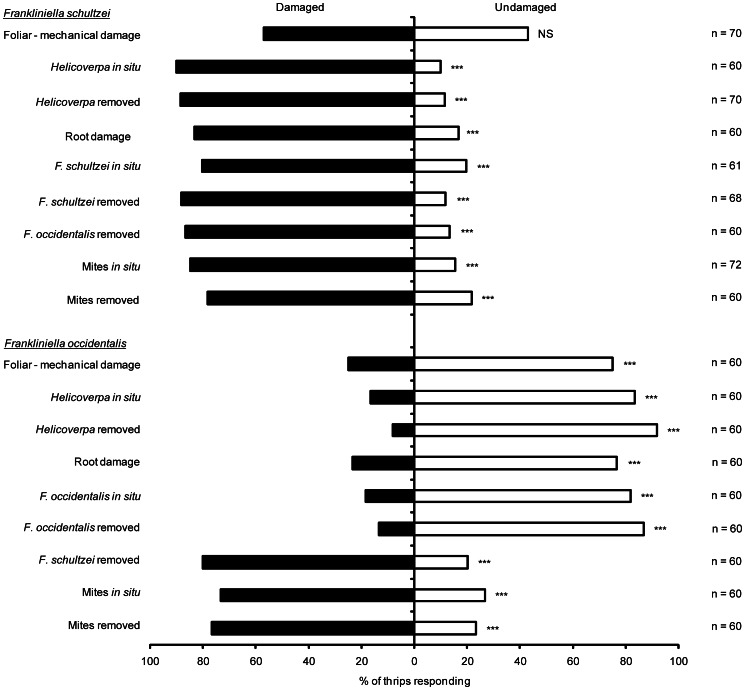
Response of two congeneric thrips species, *F.* *schultzei* and *F. occidentalis*, to various forms of herbivory inflicted on cotton seedlings. NS – not significant, *P<0.05, **P<0.01, ***P<0.001.

In general, the two congeneric thrips species responded differently to the various forms of herbivory inflicted on cotton seedlings. *Frankiniella schultzei* did not show differential attraction between mechanically damaged and undamaged plants (χ^2^ = 1.4, d.f. = 1, P = 0.2320; Figure 1). However, *F. schultzei* was more attracted to herbivore-damaged plants compared to undamaged plants, regardless of whether herbivores were *in situ* or if they had been removed (*H. armigera in situ*: χ^2^ = 38.4, d.f. = 1, P<0.0001; *H. armigera* removed: χ^2^ = 40.6, d.f. = 1, P<0.0001; root damage: χ^2^ = 26.6, d.f. = 1, P<0.0001; thrips (*F. schultzei* ) *in situ*: χ^2^ = 22.4, d.f. = 1, P<0.0001; thrips (*F. schultzei* ) removed: χ^2^ = 39.8, d.f. = 1, P<0.0001; thrips (*F. occidentalis*) removed: χ^2^ = 32.2, d.f. = 1, P<0.0001; mites *in situ*: χ^2^ = 34.8, d.f. = 1, P<0.0001; mites removed: χ^2^ = 19.2, d.f. = 1, P<0.0001; Figure 1).

Conversely, *F. occidentalis* was more attracted to undamaged plants than to damaged plants, regardless of whether the damage was mechanical (χ^2^ = 15.0, d.f. = 1, P = 0.0001) or whether herbivores were *in situ* or had been removed (*H. armigera in situ*: χ^2^ = 26.6, d.f. = 1, P = 0.0001; *H. armigera* removed: χ^2^ = 41.6, d.f. = 1, P<0.0001; root damage: χ^2^ = 17.0, d.f. = 1, P<0.0001; thrips (*F. occidentalis*) *in situ*: χ^2^ = 24.1, d.f. = 1, P<0.0001; thrips (*F. occidentalis*) removed: χ^2^ = 32.2, d.f. = 1, P<0.0001; Figure 1). However, when exposed to *F. schultzei* (removed) damaged cotton seedlings, *F. occidentalis* was more attracted to damaged plants compared to undamaged plants (χ^2^ = 21.6, d.f. = 1, P<0.0001; Figure 1). With respect to responses to mite damaged plants, *F. occidentalis* behaved similarly to *F. schultzei* in that mite-damaged seedlings were more attractive than undamaged seedlings (mites *in situ*: χ^2^ = 13.0, d.f. = 1, P = 0.0003; mites removed: χ^2^ = 17.0, d.f. = 1, P<0.0001; Figure 1).

### Degree of Foliage Damage and Thrips Attraction

**Figure 2:**
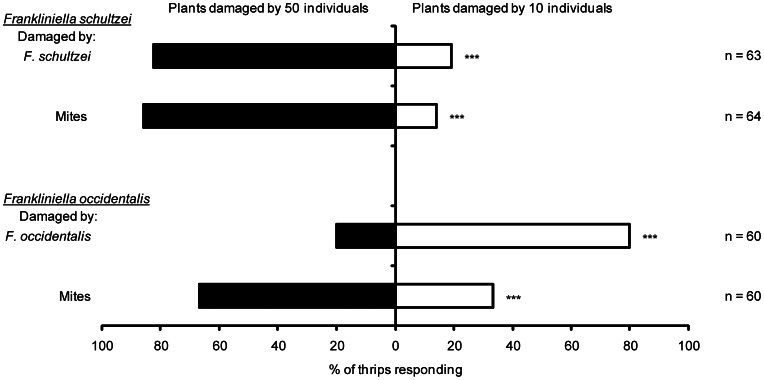
Quantitative relationship between the number of individual thrips or mites damaging cotton seedlings and subsequent thrips attraction to seedlings. Response of *F. schultzei* (above) and response of *F. occidentalis* (below). NS – not significant, *P<0.05, **P<0.01, ***P<0.001.

The degree of damage inflicted by insect herbivores significantly affected the level of attractiveness to both thrips species. Significantly more *F. schultzei* were attracted to plants damaged by 50 thrips (*F. schultzei*) than to plants damaged by 10 thrips (*F. schultzei*) (χ^2^ = 25.0, d.f. = 1, P<0.0001; Figure 2) and to plants damaged by 50 mites than to plants damaged by 10 mites (χ^2^ = 33.0, d.f. = 1, P<0.0001; Figure 2). However significantly more *F. occidentalis* were attracted to plants damaged by 10 thrips (*F. occidentalis*) than to plants damaged by 50 thrips (*F. occidentalis*) (χ^2^ = 21.6, d.f. = 1, P<0.0001; Figure 2) whereas significantly more *F. occidentalis* were attracted to plants damaged by 50 mites than to plants damaged by 10 mites (χ^2^ = 6.66, d.f. = 1, P = 0.0098; Figure 2).

### Mite Damage vs Thrips Damage on Thrips Attractancy

**Figure 3:**
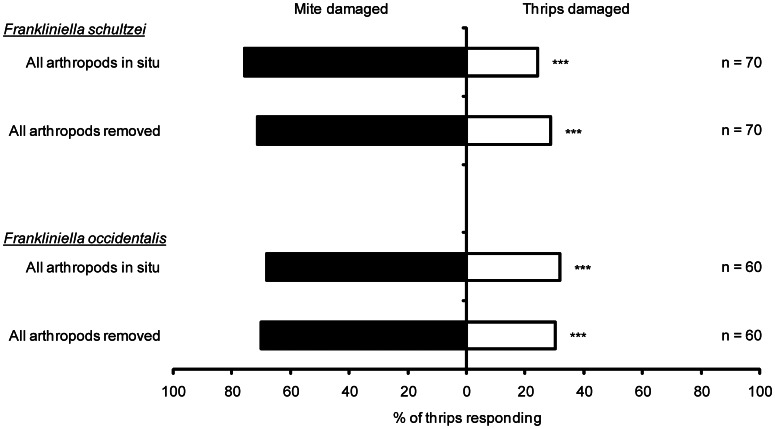
Response of thrips to either mite damaged or thrips damaged cotton seedlings. Response of *F. schultzei* (above), response of *F. occidentalis* (below). NS – not significant, *P<0.05, **P<0.01, ***P<0.001.

Both thrips species behaved similarly when presented with mite-damaged and thrips-damaged plants simultaneously. Significantly more *F. schultzei* were attracted to mite damaged plants than thrips damaged plants regardless of whether the arthroponds were present or had been removed (all arthropods *in situ*: χ^2^ = 18.5, d.f. = 1, P<0.0001; all arthropods removed: χ^2^ = 12.8, d.f. = 1, P = 0.0003; Figure 3). Similarly, significantly more *F. occidentalis* were attracted to mite damaged plants than to thrips damaged plants (all arthropods *in situ*: χ^2^ = 8.06, d.f. = 1, P = 0.0045; all arthropods removed: χ^2^ = 9.60, d.f. = 1, P = 0.0019; Figure 3).

### Are Thrips Attracted to Mites alone (No Plants) or a Combination of Mites and Damaged Plants?

**Figure 4:**
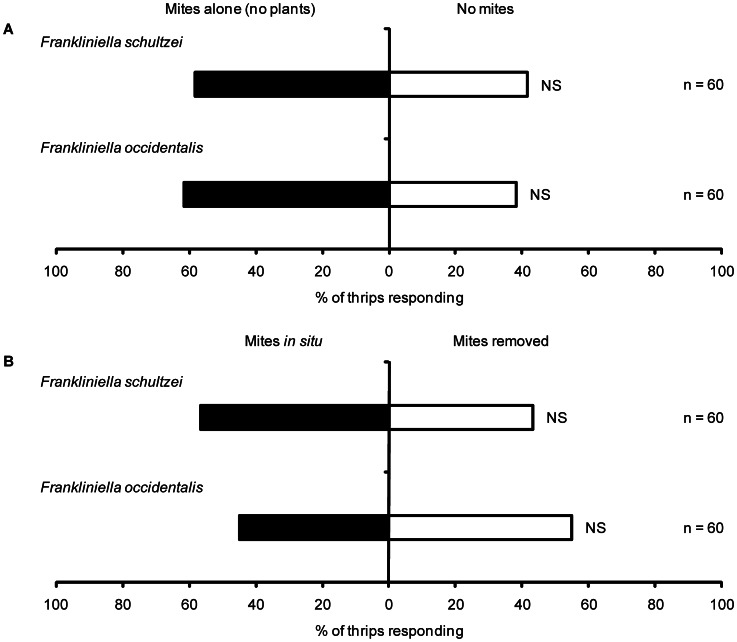
Response of thrips to mites alone (no plants) or a combination of mites and damaged plants. (A) Response of *F. schultzei* and *F. occidentalis* to mites alone (B) Response of *F. schultzei* and *F. occidentalis* to mite damaged plants from which mites were removed and on plants with mites *in situ*. NS – not significant.

Both species behaved similarly when presented with mites in the absence of plants and mite-damaged plants. *Frankiniella schultzei* was not attracted to mites when they were presented in the absence of plants (χ^2^ = 1.66, d.f. = 1, P = 0.196) (Figure 4A) and it did not discriminate between mite damaged plants from which mites had been removed and plants on which mites remained *in situ* (χ^2^ = 1.06, d.f. = 1, P = 0.30) (Figure 4B). Similarly *F. occidentalis* was not attracted to mites when they were presented in the absence of plants (χ^2^ = 3.26, d.f. = 1, P = 0.0707) (Figure 4A) and it did not discriminate between mite damaged plants from which mites had been removed and plants on which mites remained *in situ* (χ^2^ = 0.60, d.f. = 1, P = 0.4386) (Figure 4B).

## Discussion

### Specifics and Generalizations about the *Frankliniella* - Cotton System

We expected that both thrips species would show similar responses to induced changes in cotton plants and predicted that they would be attracted to plants damaged by mites but avoid plants damaged by other herbivores. However, the species responded quite differently, but consistently, to damage inflicted on cotton seedlings. *Frankliniella schultzei* was consistently attracted to cotton seedlings damaged by all herbivores tested including conspecifics, but not to plants damaged mechanically (Figure 1). In contrast, *F. occidentalis* was more strongly attracted to undamaged cotton seedlings than to mechanically damaged seedlings or to seedlings damaged by conspecifics, *H. armigera* or *T. molitor* but was attracted to seedlings damaged by *F. schultzei* or mites (Figure 1). The prediction that thrips would be attracted to plants damaged by mites, was thus correct and similar for both species. For *F. occidentalis* this result contradicts expectations based on its responses to plants damaged by other herbivores, but is consistent with previous work investigating its responses to mite damaged cotton plants [30]. Hence, the responses of both species of thrips to damage induced in host plants is clearly context dependent but unpredictable. Understanding the molecular and biochemical changes in plants and their perception by thrips will be essential to fully understand these herbivore-host plant interactions. Two particular results further highlight the complexity of the interactions between cotton and these two species of thrips. One, when given a choice between undamaged seedlings and seedlings damaged by *F. schultzei*, *F. occidentalis* was much more strongly attracted to *F. schultzei* damaged seedlings (Figure 1). This was surprising as *F. occidentalis* would be expected to respond in the same manner as it did when seedlings were damaged by conspecifics (Figure 1). Two, in the quantitative test to determine the relationship between the degree of damage and thrips’ response, the responses of both species were related to density (Figure 2) but in opposite directions; increased damaged caused by higher densities of conspecifics increased attraction of *F. schultzei* whereas the opposite was observed for *F. occidentalis*.

Thus insect herbivore-induced responses should not be solely viewed as plant defence strategies; by using the term ‘defences’, we automatically infer that plants are resisting various forms of herbivory. In cotton, responses induced by mites may be a defence against further mite attack yet the same responses are evidently used by thrips as a cue for the presence of mite prey. Further, cotton seedling responses to damage by other foliage or root feeders resulted in the avoidance of these plants by *F. occidentalis* and attraction to them by *F. schultzei*. Although induced responses may be interpreted as direct or indirect defence mechanisms, they are perhaps more parsimomously interpreted as biochemical responses to herbivory with the resultant volatiles being utilized in different ways by different species.

The intricacy and reciprocity of the interactions that we observed between the two thrips species and cotton seedlings highlights just how difficult it is to generalize about the way in which these particular organisms respond to induced plants, and the results presented here add to the variability of interactions that have been recorded between insects and plants (Table 1). In the introduction we question the validity of broad scale conclusions drawn by extrapolation from specific studies. From the current study it could be argued that *F. occidentalis* was more attracted to undamaged plants over herbivore damaged plants as a result of induced resistance. However De Vries et al. [34] showed that *F. occidentalis* damaged bean and cucumber plants were more attractive to conspecific females than undamaged plants. This provides further evidence for the context, or system, specificity of such interactions and highlights the danger of broader scale interpretation without specific testing. Further adding to the complexity, the current study shows that the responses of one host plant to herbivory had opposing effects on two congeneric insect herbivores. Other studies have shown that multiple host plants have opposing effects on a single insect herbivore species [12], [19]. For instance, the diamondback moth (*Plutella xylostella*) laid more eggs on Jasmonic acid (JA) treated cabbage plants and fewer on (JA) treated Chinese cabbage, however *Pieris rapae* did not show such a differential response between JA treated and control cabbage and Chinese cabbage plants [19]. Thus extrapolation from specific studies needs to be viewed with caution, and conclusions should be viewed in a context dependent manner.

### Novelties about the *Frankliniella* -cotton System

A challenge in trying to understand the responses recorded in this system is to determine what exactly the thrips are responding to and why they do so. The current study provides clear evidence that both study species respond to herbivore induced changes in the host plant rather than the presence of herbivores themselves (Figure 1 and 4). Previous studies on the interaction between *F. occidentalis* and cotton have also found that these thrips are attracted to undamaged cotton plants [35]–[38]. The current study is the first to investigate the interaction between *F. schultzei* and its cotton host plant. The only response that was consistent across the two thrips species was that they were attracted to mite-damaged seedlings (Figure 1 & 3). A possible reason for this is that *F. schultzei* and *F. occidentalis* are predators of mite eggs [27]. Feeding studies have shown that omnivory helps to meet the nutritional needs of thrips [37], [31] and, it has been argued that induced responses of host plants influence *F. occidentalis* to prey more on spider mite eggs and less on cotton leaves; essentially induced responses to herbivory indirectly cause omnivorous thrips to increase predation of mites [37], [38].

This argument follows the nomenclature that plant induced responses constitute a defence strategy. Induced responses to herbivory brought on by mites can both directly and indirectly reduce mite populations causing omnivores to increase predation and enhance their role in biological control strategies [37]. These generalizations must be taken with caution, as results of the current study show that *F. schultzei*, which is also an omnivore, was attracted to damaged cotton seedlings regardless of whether mites or other arthropods had caused the damage. Mite damage possibly induces different responses in the plant to the other insect species tested, hence both thrips species may be attracted to indications of the presence of mites (potential prey) but have different orientations to indications of damage by other herbivores. A mechanistic explanation is potentially available because plant transcriptional responses can be specific to the elicitor compounds released by particular insect (or mite) herbivore feeding [39], [40]. These herbivore specific plant responses to feeding could be caused by a combination of factors resulting from herbivore specific physical damage and the type and amount of elicitors released during feeding [41]. The feeding modes or sites of *H. armigera*, *T. urticae* and *T. molitor* are very different from one another and could have induced the cotton plants in very different ways. So it is possible that thrips are not responding to mite damage alone, as *F. schultzei* individuals were attracted to other insect damaged plants (Figure 1).

The current study is also the first to demonstrate that below-ground herbivory affects the responses of thrips species to their host plants (Figure 1), with the two thrips species responding differently to root damage on cotton. Studies on other insects have shown similar results [25], [53], [12]. Induced responses brought on by root herbivory have recently been shown to result in systemic responses in the shoots [42]. The current study shows that one plant with root damage (cotton) had different effects on insect herbivores (thrips) but previous studies have shown that root herbivory on different plant species have opposing effects on a single insect herbivore [12], further illustrating the complex nature of insect-plant interactions. Interactions between below-ground and above-ground insect herbivores further complicates and only adds to the challenge in predicting induced plant responses and insect behavioural responses.

### Methodology and How it Affects Making Generalizations

An important conclusion that emerges from the current study is that methodology has important consequences when making generalizations about induced plant responses and developing hypotheses that relate to this phenomenon. The methods used will influence the validity of the test and thus the quality of any derived generalization [39]. The results presented indicate that tests should include multiple insect herbivores if a single plant is used or multiple host plant species if a single insect herbivore is tested. This will provide more meaningful tests (and answers) into how plants respond to insect damage and how insects respond to plant damage, and will avoid the problem of selecting study organisms and designing tests that are simply likely to verify earlier generalizations.
